# Infrasonic Earthquake Detectability Investigated in Southern Part of Japan, 2019

**DOI:** 10.3390/s21030894

**Published:** 2021-01-29

**Authors:** Islam Hamama, Masa-yuki Yamamoto

**Affiliations:** 1 School of Systems Engineering, Kochi University of Technology 185, Miyanokuchi, Tosayamada, Kami, Kochi 782-8502, Japan; yamamoto.masa-yuki@kochi-tech.ac.jp; 2 National Research Institute of Astronomy and Geophysics, Helwan 11421, Cairo, Egypt

**Keywords:** infrasound, earthquakes, KUT infrasound sensor network, T-phase, infrasound propagation, transmission losses

## Abstract

The Kochi University of Technology (KUT) Infrasound Sensor Network contains 30 infrasound sensors which are distributed all over Japan especially in Shikoku Island. At all infrasound stations installed with three-axis accelerometers to measure the peak ground acceleration (PGA). Many earthquakes were detected by our system after establishing of the network since 2016. In this study we will focus on all the possibilities for infrasound detection generated from earthquakes using KUT sensor network and International Monitoring system (IMS) stations for the earthquakes which were detected in southern part of Japan during 2019. As for earthquakes with strike-slip mechanisms the P-waves could not be detected by our sensors. In addition, The conversion from seismic to acoustic waves can be happened through the generating of the T-phase from oceanic earthquakes. On 9 May 2019, progressive multi-channel cross correlation (PMCC) method applied infrasound and hydroacoustic waves from two earthquakes happened in west of Kyushu Island as the T-phase was well-recorded at H11N station near Wake Island. Moreover, infrasound propagation modeling is applied to the reconstructed atmosphere profile by Ground to Space Model (AVO-G2S) to confirm the infrasound arrivals, furthermore the 3D ray tracing process and the calculations by using the transmission loss equation with normal modes and parabolic equation methods are investigated. The study confirmed the infrasound generation scenario from the T-phase of oceanic propagation.

## 1. Introduction

**Figure 1:**
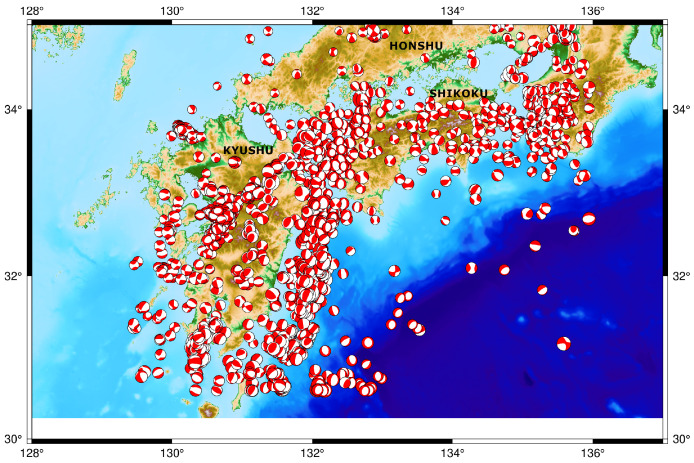
The distribution of earthquakes with different mechanisms from January 2000 to January 2020 in southern part of Japan.

Infrasound can be defined as inaudible sound waves with a frequency range from 0.003 to 20 Hz [1]. Since the historical explosion of the Krakatoa volcano occurred in 1883 the importance of infrasound increased. The displacement or rapture of the earth’s surface from the earthquakes are considered as one of the natural infrasound sources [2]. The first detection of air pressure that was generated by tectonic movements from the Alaska earthquake in 1964 (M = 8.5) was reported by Mikumo [1968] [3]. The magnitude of the earthquakes plays an important role in the infrasonic earthquake detection in case the infrasound waves can be generated by earthquakes with a magnitude higher than 5.5 mb (Body-wave magnitude) [4]. In the case of local earthquakes, the infrasound waves can be generated as a result of the ground motion due to body waves and thus well-recorded in local infrasound network [5]. In addition, the moderate shallow earthquakes with the magnitude ranges from 3 to 6 Mw (Moment magnitude) can produce infrasound waves if their topographic features are near the epicentral location such as coastal cliffs [6]. Moreover, the infrasonic signals which are resulted from earthquakes at local infrasound stations can be categorized as epicentral, local, and diffracted signals, where the arrival of the local infrasound waves are usually faster than the other types due to the nature of propagation to the receiver as seismic wave [7]. As the local infrasound waves can be generated due to the integration of the sound radiated from seismic waves, the recording of teleseismic waves in microbaroms is clear evidence of the conversion from seismic waves to sound [8]. In this study, we will focus on all possibilities for the generation of infrasound from earthquakes using a dense local infrasound network which can be near to the epicentral location of the earthquakes in the southern part of Japan during 2019. Furthermore, we will focus on the relation of the earthquake mechanisms and the detectability of P-waves in near infrasound stations. In addition, the numerical modeling of the infrasonic waves propagation was applied with the reconstructed atmospheric wind to confirm the infrasound phases which could be detected from a higher altitude of the atmosphere. The explanation of infrasound generation can follow many scenarios. If the epicentral location is near to topographic features like mountains, so the surface waves can be diffracted from these features [5]. However, if the epicenter is located in the ocean, the P-wave and the vertical polarized S-wave vibrate on the ocean floor. These vibrations are the main reasons for the generation of acoustic waves which reflected in the Sound Fixing and Ranging (SOFAR) channel and propagated as T-phase in all directions [9]. Since ocean-borne acoustic phases from earthquakes are detected below the seafloor, T-phases are high-frequency acoustic waves with large propagation distances. These phases are generated as a symmetric coda about the peak frequency arrivals. Furthermore, T-phases are generated from the seismic to acoustic coupling mechanisms [10]. Shikoku Island is close to seismic source regions along the Nankai Trough. During the last 20 years, more than 1900 earthquakes have been recorded in the southern part of Japan according to Japan Meteorological Agency (JMA and NIED, 2019) as is shown in Figure 1, distributing over Shikoku and Kyushu Islands with different mechanisms. The earthquakes over Shikoku Island can be classified based on the depth i.e., a part is with very shallow depths (lower than 20 km), and the others are deeper than 20 km. The seismic activity in the region was clearly low before the great Tonankai earthquake in 1946 [11]. The ground to air coupling also are clear in this area due to the shallow depths of recorded earthquakes. In order to understand the generation of infrasound from earthquakes, a local dense network with infrasound sensors (KUT Infrasound Sensor Network) was used to detect all possibilities for infrasonic waves as a single sensor in addition to the array of I30JP infrasound station of the International Monitoring System (IMS). This study will focus on the earthquakes recorded in the southern part of Japan during 2019. The aim of the study is to show all possible mechanisms of infrasound coupling from earthquakes with using KUT local and dense infrasound sensor network, moreover, we focused on the relation between generated T-phase from oceanic earthquakes and the generation of infrasonic waves. In addition, the infrasound propagation modeling and transmission losses from the earthquake to the stations were calculated to confirm the arrival phases at the infrasound stations.

## 2. Data Sets and Method

### 2.1. Infrasound Data

**Figure 2:**
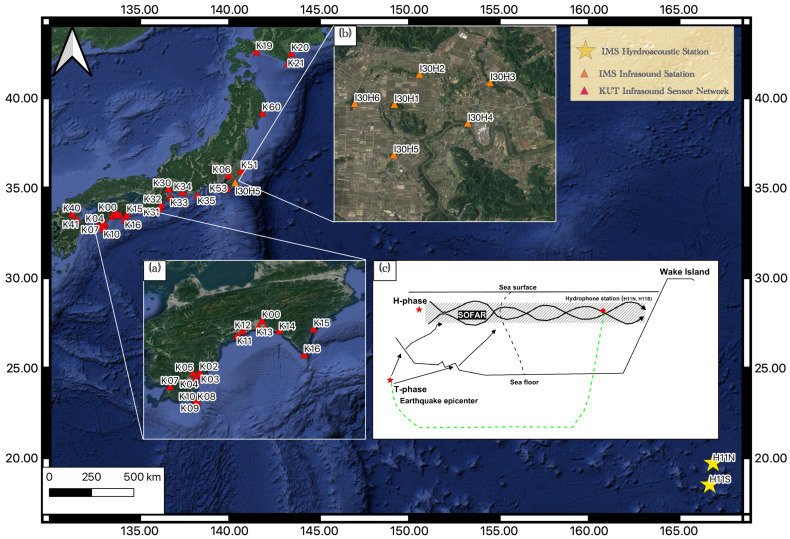
(**a**) The locations of Kochi University of Technology (KUT) infrasound sensors which are distributed in Shikoku Island, (**b**) IMS I30JP infrasound station which consists of 6 infrasound sensors with aperture varying from 1 to 3 km, (**c**) The generation and the propagation of the T-phase from the earthquake epicenter to H11N and H11S stations near Wake Island.

KUT Infrasound Sensor Network consists of 30 infrasound sensors which are distributed all over Japan, a big part of the network is distributed over Shikoku Island as shown in Figure 2a. KUT Infrasound Sensor Network started its operation in 2016 as a real-time monitoring network and it is also available in web applications and open access. KUT Infrasound sensor is a comprehensive sensor that is integrated with a three-channel accelerometer, a barometer sensor, a microphone for detecting noise level and infrasound (differential pressure) sensor [12]. In addition to the KUT network, we use the I30JP Infrasound station of IMS which consists of 6 MB-2000 infrasound arrayed sensors with the distance varying from 1 to 3 km as shown in Figure 2b.

### 2.2. Hydroacoustic Data

IMS contains 11 hydroacoustic stations which are distributed all over the world. In this study the hydroacoustic waves are extracted from H11N station which consist of 3 sensors of N1, N2, and N3. This station is located near Wake Island at about 3750 km from the earthquake epicenter locations as shown in Figure 2.

### 2.3. Infrasound Analysis Method

DTK-GPMCC program (CEA/DASE, 2018) is used to analyze the infrasound signals for array analysis. Here we use progressive multichannel cross correlation (PMCC) technique which depends on correlation between sensors that can be treated as the same signal as after applying Fourier transform: $\left(s\left(f\right)=A{\left(f\right)}^{i\varphi (f)}\right)$, where (A(f)) represents the spectral amplitude and (φ(f)) is the phase at a specified frequency band. Usually the ambient noise is characterized by quick change in both amplitude and phase. Assuming that we have two sensors, so the signal detection is valid through the following relations: $\left(A_{2}\left(f\right)=A_{1}\left(f\right)\right)$ and $\left(\varphi_{2}\left(f\right)=\varphi_{1}\left(f\right)-\Theta \left({\vec{r}}_{2}-{\vec{r}}_{1}\right)\right)$, where $\left(\vec{r_{n}}\right)\left(\left(n=1,2\right)\right)$ means sensor position vector. As the signal should be the same in different sensors in the array but the difference only in the delay between the sensors depending on their positions, the delay between the *i*-th and j-th sensors can be estimated through the following relation: $\left(\Delta t_{i}j=1/2\pi f\left(\varphi_{i}\left(f\right)-\varphi_{j}\left(f\right)\right)\right)$. For each group of three sensors ((i,j,k)), the sum of the time delays for a plane wave signal obeys a closure relation: (Δtij+Δtjk+Δtki=0). Since the advantage of PMCC technique is to reduce the false detections in the sensors, the calculations depend on the initialization of these sub-networks which consist of arrayed sensors close to each other [13]. The PMCC technique can be applied to seismic, hydroacoustic, and infrasound waves [14].

## 3. Observations

**Figure 3:**
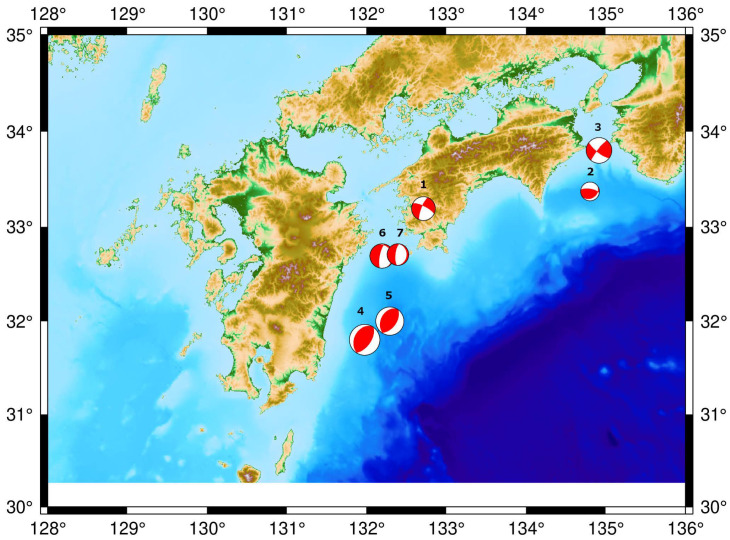
The selected 7 events in the study according to 3 parameters: magnitude, depth, and mechanisms.

Earthquake events which are chosen in this study are recorded in different seismic bulletins such as Japan Meteorological Agency (JMA), United States Geological Survey (USGS, 2019), and Reviewed Event Bulletin (REB) of International Data Center (IDC) (CTBTO, 2019). These earthquakes are with magnitudes higher than 4.4 mb and shallow depths (shorter than 50 km) with different source mechanisms as shown in Table 1 and Figure 3.

**Figure 4:**
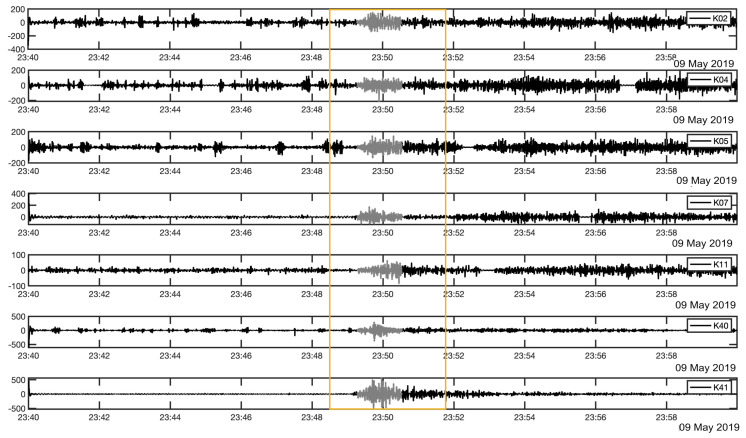
Filtered [0.2, 2] Hz waveform recorded P-waves of the earthquake occurred at 23:48:41.68 UTC on 9 May 2019 and well recorded at 7 sites of KUT Infrasound Sensor Network (K02, K04, K05, K07, K11, K40, K41). The yellow rectangle focuses on the window length of 120 s within the origin time and gray parts of the signals illustrate the clear arrival of the P-waves to the sensors in a range of 250 km from the earthquake.

The recorded infrasound waves induced from earthquakes can be classified into three categories. Firstly, the infrasound waves were recorded from the generated P-waves in near infrasound sensors less than 200 km. In this case, these records are all clear on earthquakes with normal and reverse fault mechanisms as shown in Figure 4. Secondly, the infrasound waves which are resulted from the coupling of ocean-air through the T-phase which transmits through the SOFAR layer on the ocean. Thirdly, the near topographic features which will enhance the propagation of the infrasound waves, however, if the amplitude of the earthquake is not higher than 5.5 mb it will be difficult to detect infrasound waves coming from oceanic earthquakes, meaning that not only one factor may control the infrasound coupling from earthquakes.

**Figure 5:**
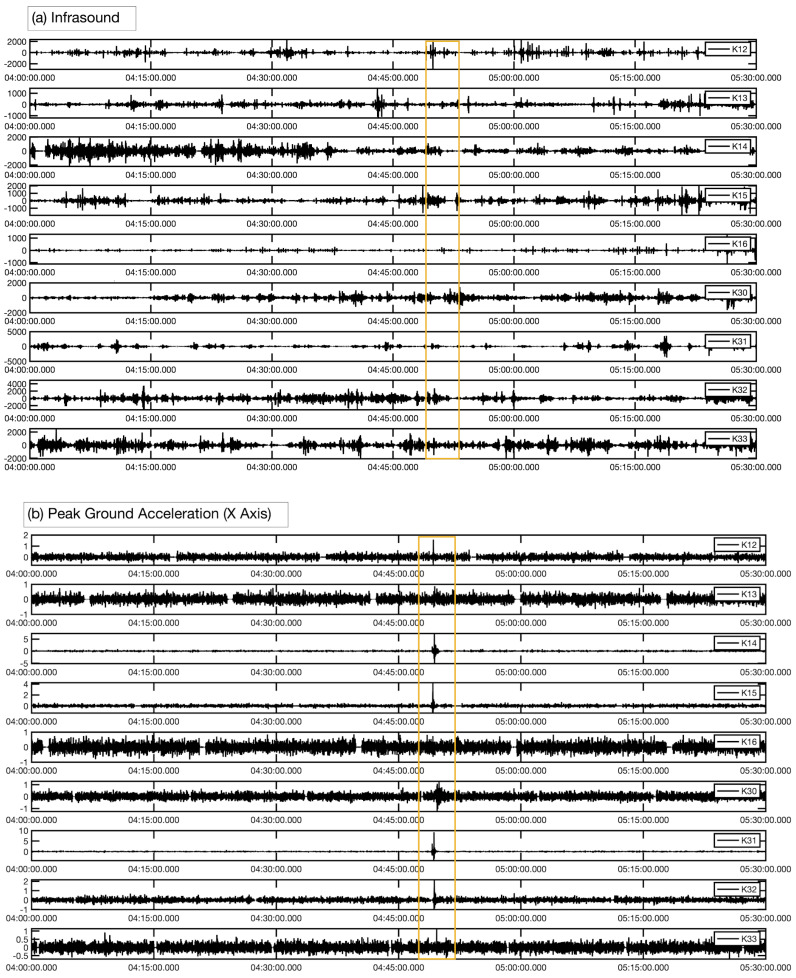
(**a**) Filtered waveform [0.2, 2] Hz shows no detection of P-waves at infrasound sensors near to the epicentral location at 04:48:48 UTC on 13 March 2019, (**b**) The recording of P-Waves in accelerogram from the same sensors shows the clear detection of P-waves. The yellow rectangle focuses on the window length for expecting arrivals of P-waves on both infrasound and accelerograms.

Not likely, in the earthquakes which are generated from strike-slip fault mechanism, no P-waves were detected by infrasound sensor as shown in Figure 5, for sensors very close to the epicentral locations in the cases of 11 and 13 of March 2019. As the signals from near infrasound sensor did not well-correlated in time and frequency domain, moreover, among many studies applied on the generation of infrasound from earthquakes, only a few of them reported with a super shear-rupture speed [15]. According to Shelly et al. (2008) [16] Shikoku Island is characterized by low-frequency earthquakes and thus, one possibility that the tremor results from a slow shear slip. This may be a reason for making the small detectability of P-waves near a local station in Shikoku Island. The infrasound coupling from seismic waves for long distances occurred in two recorded earthquakes on 9 May 2019.

**Figure 6:**
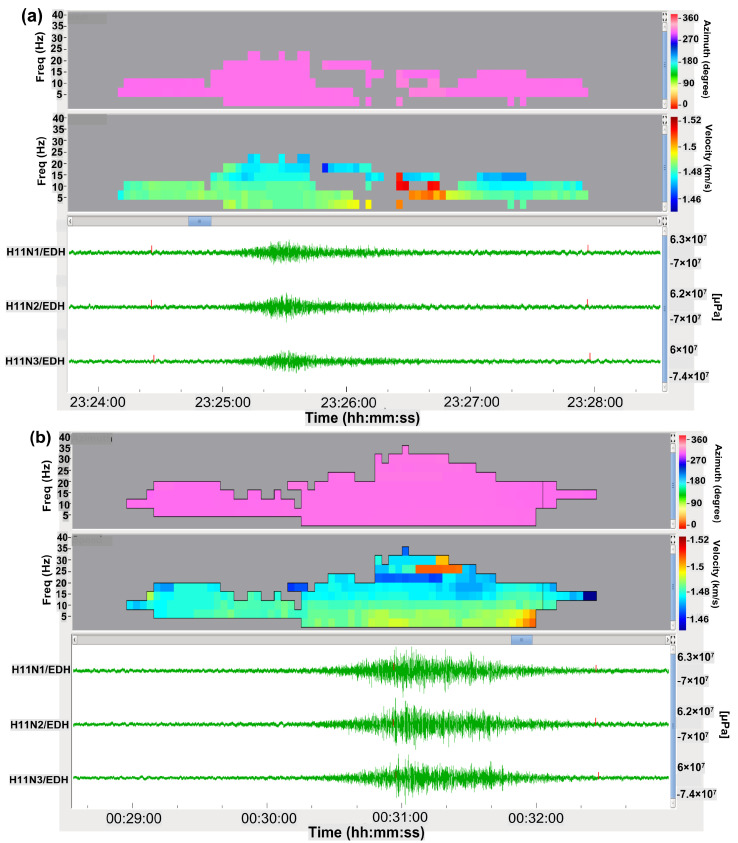
(**a**) Long duration T-phase waves which were recorded from the foreshock which occurred at 22:43:21 UTC at H11N station between 23:24:30 and 23:28:00. (**b**) Long duration T-phase waves which were generated from the main shock at 23:48:41 UTC and were recorded at H11N after almost 1 hour from the previous record between 00:29:00 and 00:32:40.

According to the observations as well as the previous report [4], the ground-atmosphere or ocean-atmosphere coupling could take place in earthquakes with magnitudes higher than 5.5 mb. In this study, the focal point was two earthquakes which were recorded on 9 May 2019 (UTC) at 22:43 and 23:48 according to JMA, successive events with the maximum magnitude during 2019 in the southern part of Japan. Their epicentral locations were in the oceanic region and they are also classified as shallow earthquakes with normal fault mechanism which the P-waves are obviously recorded in many stations of the KUT sensor network as shown in Figure 1. In addition, to understand the nature of the generated infrasonic waves from earthquakes and the coupling criteria, hydroacoustic waves are used to clarify the mechanism of the seismic conversion to acoustic waves. H11N and H11S stations recorded T-phase from both earthquakes at the same distances of 3750 km at Wake Island as shown in Figure 6.

**Figure 7:**
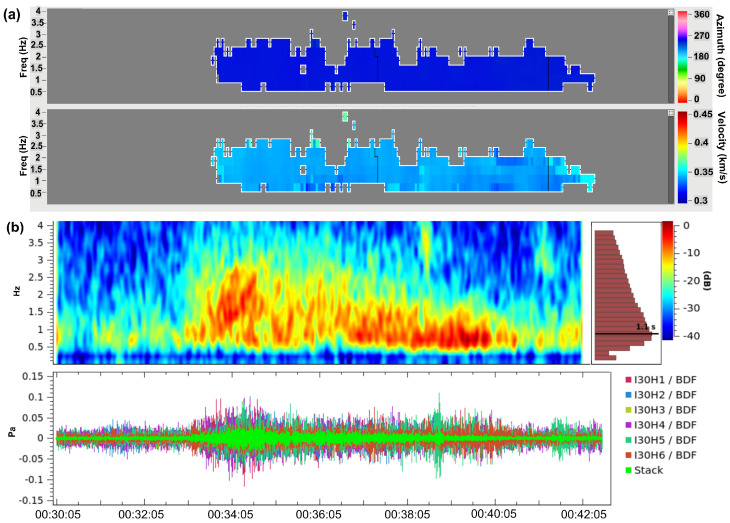
(**a**) Progression of multichannel cross-correlation for one-hour waveform from I30JP with a window length of 30 s with overlapping of 90 s at 00:31:30 UTC with a back-azimuth of 244 degrees and the frequency content range from 0.6 to 2.6 Hz with an apparent velocity of 339 m/s. (**b**) Beamforming result of the mainshock showing the increase of the signal to noise ratio from the 6 sensors of I30JP.

The hydroacoustic waves have a low-frequency content (T-Phase) ranging from 2.095 to 34.015 Hz with a speed of 1.48 km/s and back azimuth of 298.6 degrees with a duration of 200 s. These waves propagated in the SOFAR channel. In this layer, as the sound waves can travel with the lowest speed between the speed of the higher and lower layers, it can be considered as trapping with a sound speed in the ocean and it is very effective to transport the pressure for long distances. As a result of the coupling between sea and atmosphere, it becomes possible and infrasound waves recorded in the I30JP of IMS and K53 station of KUT sensor network as shown in Figure 7.

**Figure 8:**
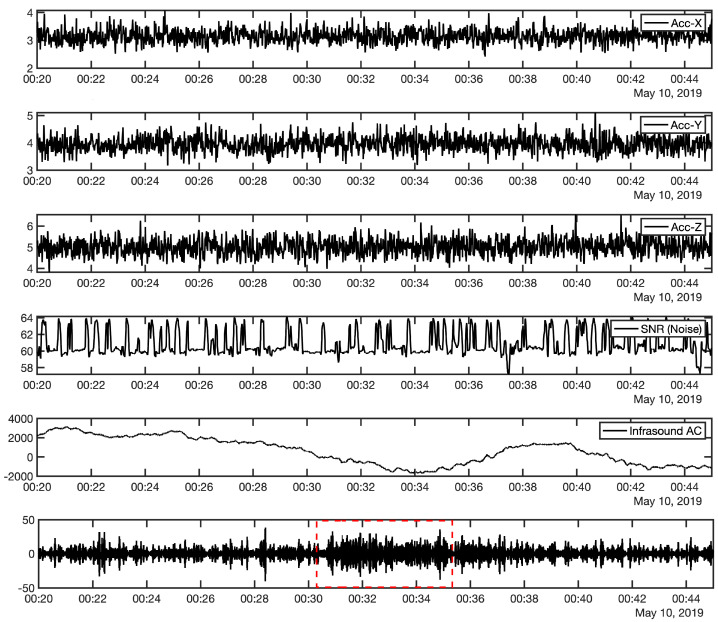
K53 comprehensive sensor recording from 00:20:00 to 00:45:00 UTC shows the same signal recorded in I30JP at 00:31:00 UTC (filtered between 1.0 and 2.0 Hz and resampled by five sample/s). The red dotted rectangle is the window of the detected infrasonic signal.

The K53 station is near to the I30JP array and detected the same infrasonic kind of wave from the mainshock as shown in Figure 8, the recorded signal amplitude in the site of K53 and I30H3 weaker than the other five sensors of I30JP, the reason may be due to the location of K53 near the coastal line and the data affected by the ocean waves, furthermore, in I30H3 site it may be due to some differences in the noise-reduction system there; however, K53 can be combined with I30JP array to calculate the azimuth and the apparent velocities from the cross-correlation process in the frequency domain.

**Figure 9:**
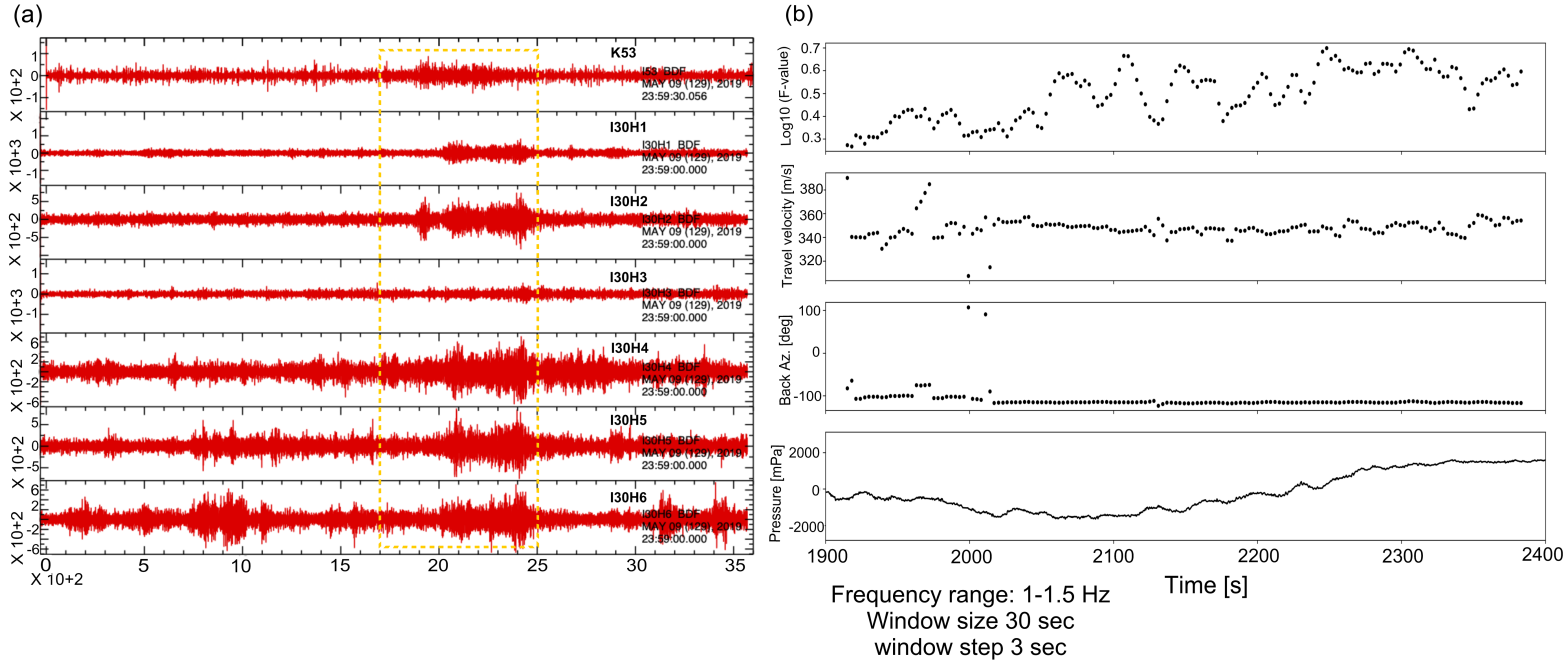
(**a**) Infrasonic waves recorded from I30JP sensors and K53 together, yellow dot rectangular is a focused window of the infrasound arrival. (**b**) Beamforming of the sensors of IMS I30JP and single sensor of KUT K53 for one hour from 23:00 to 00:00 UTC with the F-ratio getting higher at a trace velocity of about 340 m/s and an azimuth of—117 degrees.

By applying the beamforming method using Infrapy package [17] to the I30JP with K53 station, the results are well correlated with the previous array analysis of I30JP alone as shown in Figure 9, moreover, the arrival of K53 is about 150 s earlier than that of I30JP as the difference in distance between them. As a result, the correlated waves have a mean apparent speed of 340 m/s and a frequency range between 0.6 and 2.6 Hz, with a mean back-azimuth of 242 degrees. The infrasound waves which coupled from P-waves characterized by high travel velocity ranges from 5 to 8 km and short time duration, furthermore it can be detected by near infrasound sensors. However, the infrasound waves which are generated from the T-phases can be identified by long time duration and it can be predicted according to the nature of the infrasound propagation during the source time, in addition, these signals usually are recorded in near infrasound sensors from the oceans.

## 4. Infrasound Propagation Analysis and Discussion

**Figure 10:**
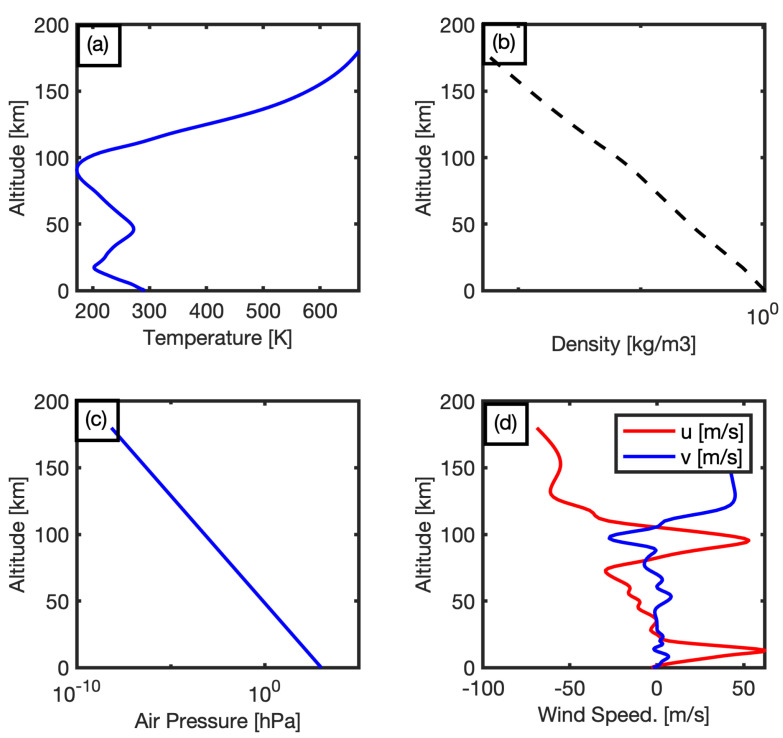
(**a**–**c**) The computed temperature, density, and air pressure from 0 to 200 km using Naval Research Laboratory Mass Spectrometer and Incoherent Scatter Radar, Exosphere (NRL-MSISE) Model, (**d**) the horizontal wind speed profile which was estimated from AVO-G2S model. The red line refers to the zonal (eastward) wind speed (u) in m/s and the blue line refers to the meridional (northward) one (v).

In general, classical ray-tracing is an infrasound propagation modeling that assumes the linear propagation on a plane parallel to the acoustic wave-front as described by Groves [1955a] [18]. For local propagation calculations, the normal mode technique is much effective, which it assumed that the vertical wind speed can be neglected [19]. The parabolic equation technique solves many problems in cases of range-dependent propagation. This method was previously not effective for the acoustic atmospheric propagation due to the low Mach number and narrow propagation angle, however, with using the wide-angle and high-Mach number condition, the parabolic equation becomes more effective without having limitations in atmospheric propagation [20]. Range-independent and range-dependent propagation methods are applied for the source to receiver simulation [21]. Reanalysis data of National Centers of Environmental Prediction (NCEP) with a resolution of 2.5 degrees is integrated with Horizontal Wind Model 14 (HWM-14) [22]. As well as Naval Research Laboratory Mass Spectrometer and Incoherent Scatter Radar, Exosphere (NRL-MSISE) [23] are used for reconstruction of horizontal wind speed profile with AVO-G2S package [24] which integrates real analysis data together with modeling data, high-resolution profile which can reach to the upper atmosphere. NCEP reanalysis data and HWM-14 provide the data of horizontal wind speed (Meridional, Zonal), furthermore the NRL-MSISE model generates temperature, density, and air pressure. The estimated atmospheric wind profile above the epicentral location of the earthquake at 23:48:41 UTC on 9 May 2019 is shown in Figure 10.

**Figure 11:**
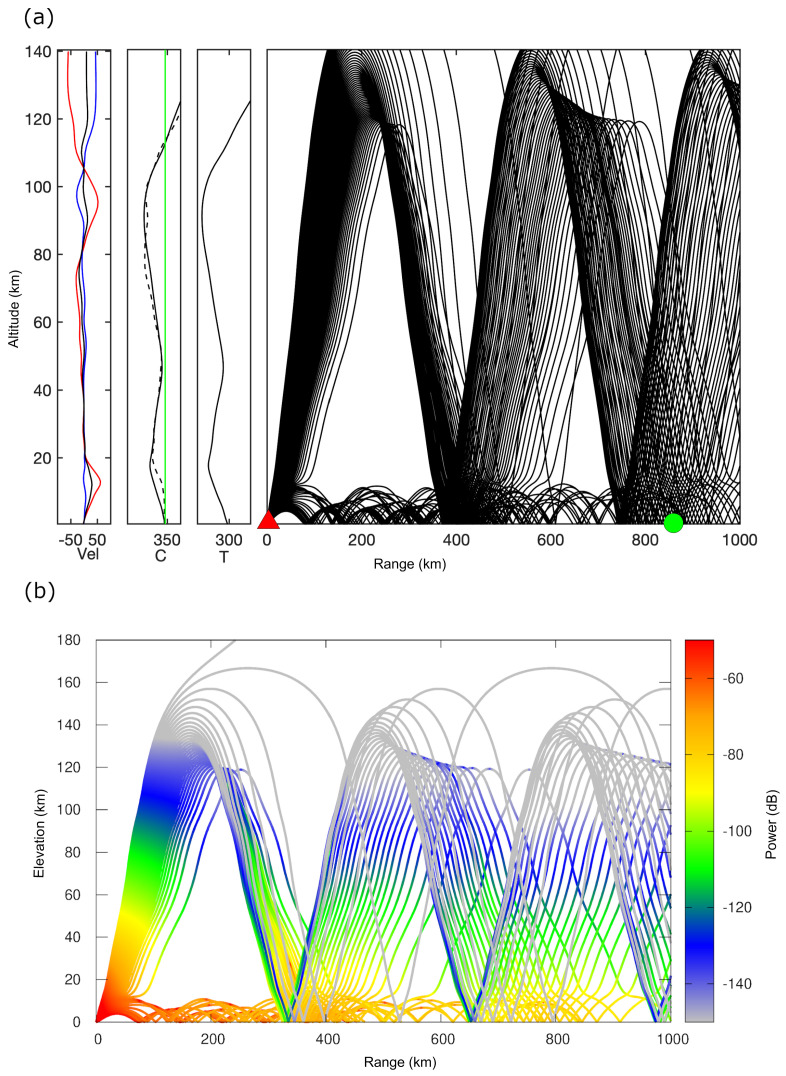
(**a**) The profile resulted from 3D-ray tracing using stratified propagation method, from the left represents the horizontal wind speed profile, sound speed, temperature, and the rays of propagation, respectively. The green circle refers to the distance from the earthquake epicenter location and I30JP. (**b**) 3D-ray tracing from the epicenter location of the earthquake to a range of 1000 km with a power of attenuation of the rays in dB.

Figure 10 shows that the wind direction in the troposphere is eastward with a speed range from 0 to 23 m/s at 0 to 20 km altitude. Moreover, the temperature, density, and air pressure are decreasing gradually. On the other hand, for the stratosphere, the temperature has a significant increase between 20 to 50 km (up to the stratopause) and reached almost the start point again, but at an altitude range from 50 to 95 km in the mesosphere the temperature has a decreasing trend. In addition to the dramatic decreasing density and air pressure, the wind direction is almost constant toward the west. Conversely, in the thermosphere, the temperature reached the peak, as well as zonal wind speed increases in the east direction at the upper troposphere and the westward jets increase above 120 km in the thermosphere. The wind profile is the main input for the simulation of infrasound propagation. Assuming the stratified atmosphere, the typical 3D-ray tracing is applied to show all the possibilities of infrasound propagation on the azimuth of 61 degrees (the azimuth to I30JP, and K53), depending on the calculation of the effective sound speed, as shown in Figure 11a.

**Figure 12:**
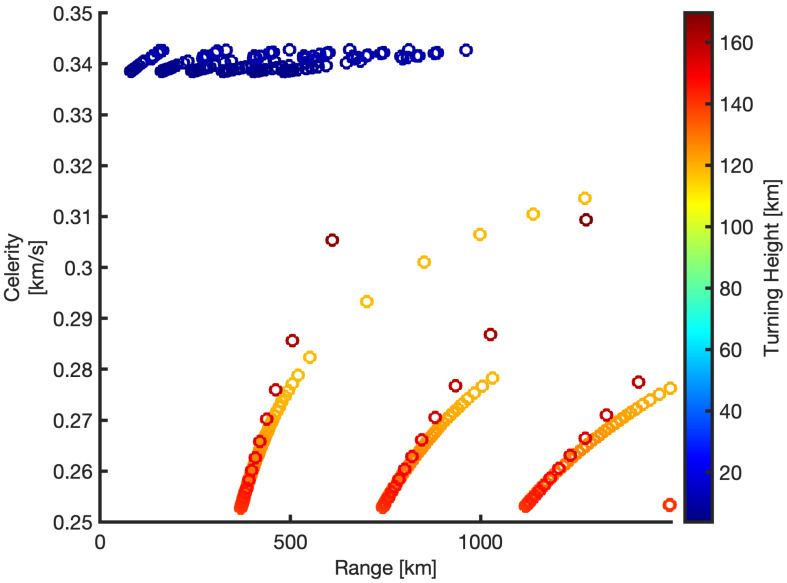
Estimated celerity within a range to 1000 km and with varying altitude from 0 to 170 km. The blue circles refer to the tropospheric phases with celerity around 340 m/s, the red to orange circles show the thermospheric phases with lower celerity.

The ducting propagation happens when the effective sound speed becomes larger than the theoretical sound speed and adiabatic sound speed, as is shown in Figure 11b. The ducting occurs obviously in the troposphere and at the lower thermosphere at about 120 km altitude, the strong jet wind refers to the eastward direction in tropospheric phases. Furthermore, the calculated travel time from the source to the station as well as the calculated celerity predicts infrasound waves with a speed of 338 to 343 m/s (tropospheric phases) coming from the lower altitudes. In the lower thermosphere, there are the other phases with celerity ranges of 225.5 to 228 m/s at the upper atmosphere as shown in Figure 12.

**Figure 13:**
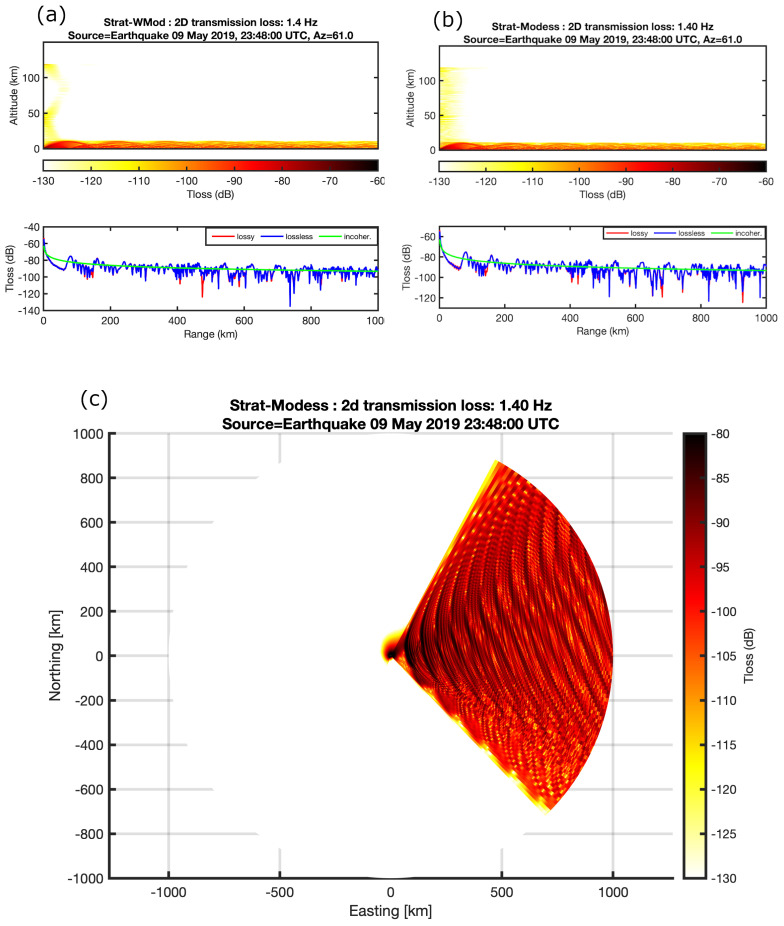
(**a**) The 1D and 2D transmission losses using wide angle high mach modal (Wmod) at a frequency of 1.4 Hz to an azimuth of 61 degrees within a range of 1000 km. (**b**) The 1D and 2D transmission losses using Modal effective sound speed (Modess) at frequency 1.4 Hz to an azimuth of 61 of degrees within a range of 1000 km. (**c**) The propagation simulation in all azimuth distribution from 0 to 360 degrees.

Transmission loss calculations are applied to confirm the infrasound propagation from the source to the receiver and the accuracy of the ray tracing. Firstly, the range-independent propagation routines, such as Normal Modes and Wide-Angle High Mach Modal (WMod) are used to calculate the power transmission of the infrasound waves from the epicentral location to I30JP which recorded the event. As the main difference between the two routines is that the WMod does not depend on the effective sound speed rather than the wind profile with assuming the vertical wind shear can be very small compared with the wavelengths, it is much better if the background of wind speed is relatively high. The transmission loss calculations are similar by the two routines at 1.4 Hz which is the mean recorded frequency of the event at I30JP. However, the results from the two routines at a frequency of 1.4 Hz are similar as shown in Figure 13a,b, moreover, the transmission losses were calculated for all azimuth directions from the source to 1000 km distance as shown in Figure 13c, the losses of 1.4 Hz signals in the north–east to south–west direction are almost lower than 105 dB which showed the all possible directions of the infrasound propagation.

**Figure 14:**
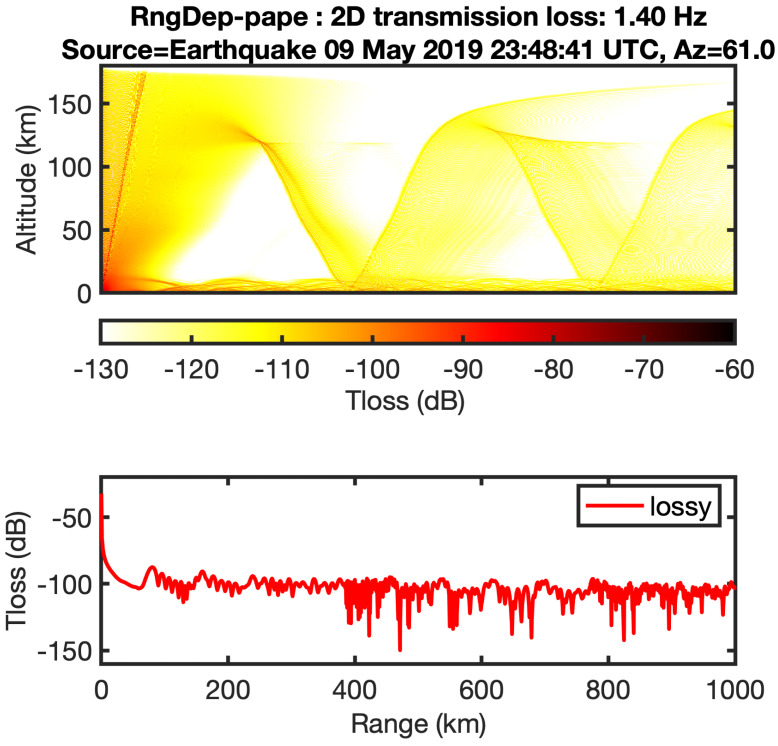
The power transmission loss for infrasound waves from the source to the receiver using parabolic equation.

Based on the results derived by two routines, arrivals of infrasound waves from the troposphere are confirmed only for a distance of 870 km. As the estimated transmission losses in signals are lower than 100 dB in the direction to the infrasound sensors. The range-dependent propagation based on the parabolic equation algorithms is applied for making a comparison between range independent and range-dependent techniques, assuming that the ground impedance is rigid with using the same frequency of 1.4 Hz. In this method the atmosphere is assumed as a grid of vertical profiles and no longer stratified, thus the transmission loss calculations imply the presence of ducting from the troposphere and thermosphere at the station location. The power of transmission for the troposphere phases is slightly higher than the thermosphere phases at long distant propagation as shown in Figure 14.

By comparing the predicted arrivals between the range-dependent and the range-independent methods with the real data, the arrival of tropospheric phases. The tropospheric arrivals at a distance of 870 km are confirmed by both results. Furthermore, the tropospheric phases celerity is usually higher than the celerity of stratospheric and thermospheric phases (<320 m/s), as the arrival at station I30JP is 00:32:30 UTC after 2629 s from the original time of the earthquake, so the celerity of detected signals is 330 m/s which is well-correlated with predicted celerity of 338 m/s, however, the arrivals of thermospheric phases could not be confirmed in the real record of the earthquake due to the high uncertainty of wind speed at higher altitude.

## 5. Conclusions

In 2019, many earthquakes were recorded in the southern part of Japan. The KUT Infrasound Sensor Network distributed all over Japan especially concentrating in Shikoku Island which is near to the epicentral locations. Our single sensor usually recorded the arrival of P-waves as the sensor is comprehensive with the accelerogram, confirming these waves for a short distant propagation. Additionally, the IMS I30JP infrasound array located in Chiba prefecture can be used with the widely distributed KUT single sensors so as to obtain high accuracy results. In this study we checked all the recorded earthquakes which are reported in international events bulletins, seven shallow earthquakes were chosen to study the nature of infrasound waves recorded in the KUT Sensor Network and IMS I30JP station, according to the body waves magnitude and their mechanisms. For earthquakes with strike-slip mechanisms the P-waves could not be detected by our sensors, however, it is near to the epicentral location and with enough magnitude to converse the seismic waves to sound. Conversely, the earthquakes for normal and reverse mechanisms of the P-waves were recorded well near each local sensor. Furthermore, according to the observations, the earthquakes with higher than 5.5 mb generate infrasound waves over a long distance and was recorded for a distance range from 820 to 870 km in I30JP and K53, respectively, for the earthquake that occurred at 23:48:41 UTC on 9 May 2019. The coupling from the ocean to the atmosphere is confirmed by the detection of T-phase in the hydroacoustic station in the vicinity of Wake Island. As the T-phase propagated for long distances through the SOFAR layer in the ocean, the H11N hydroacoustic station clearly recorded the T-phase which may be a result of the conversion from seismic waves to sound. The forward propagation modeling from the epicentral location to IMS I30JP is applied to verify the detection, where the wind profile depends on both reanalysis radiosonde data from NCEP and the numerical model HWM-14 which enhances the horizontal wind speed profile and filling the gap for the upper atmosphere data using AVO-G2S modeling at above the event location. Range-independent and range-dependent techniques are applied to estimate the propagation rays for infrasound waves. The existence of tropospheric phases at I30JP and K53 were confirmed. However, the parabolic equation method showed weak thermosphere phases for various ranges. According to the results of transmission loss calculations obtained from different techniques, the range-independent and range-dependent propagation methods reflect the same tropospheric arrival to the receivers.

## Figures and Tables

**Table 1:** The details of selected earthquakes in the study.

No.	Date	Event Time UTC	Mag. (mb)	Lon. E	Lat. N	Depth (km)	Source Mechanism
1	7 Mar 2019	02:20:34.57	4.4 mb	134.79	33.3703	31.61	Reverse
2	11 Mar 2019	06:37:51.27	4.9 mb	132.71	33.1903	38.13	Strike-Slip
3	13 Mar 2019	04:48:48.78	5.1 mb	134.91	33.8010	43.05	Strike-Slip
4	9 May 2019	22:43:21.23	5.8 mb	131.99	31.7850	25.35	Reverse
5	9 May 2019	23:48:41.68	6.0 mb	131.97	31.8012	25.46	Reverse
6	10 May 2019	23:59:40.05	5.0 mb	132.29	32.6903	36.34	Normal
7	12 May 2019	06:07:43.73	4.7 mb	132.29	32.7053	36.66	Normal

## Data Availability

I30JP and H11N stations of IMS datasets are available for national data center members through the following website: https://swp.ctbto.org/. In addition, KUT Sensors Network datasets can be viewed through the following link: http://infrasound.kochi-tech.ac.jp/infrasound/graph.php, also the datasets can be downloaded under permission of Masa-yuki Yamamoto.
